# Multiple Roles of the Cytoskeleton in Bacterial Autophagy

**DOI:** 10.1371/journal.ppat.1004409

**Published:** 2014-11-20

**Authors:** Serge Mostowy

**Affiliations:** Section of Microbiology, MRC Centre for Molecular Bacteriology and Infection, Imperial College London, London, United Kingdom; University of North Carolina at Chapel Hill School of Medicine, United States of America

## Introduction

Several bacterial pathogens are targeted by macroautophagy (referred to as autophagy hereafter), and autophagy is widely recognized as an important antibacterial defence mechanism [1]. On the other hand, new work has shown that some bacterial pathogens have mechanisms to avoid or exploit the autophagy machinery for intracellular survival [2]–[4]. Strikingly, the host cytoskeleton plays a crucial role in autophagy and its ability to restrict or promote bacterial replication. A complete understanding of autophagy-cytoskeleton interactions is therefore needed to enable the manipulation of autophagy for therapeutic purposes. Actin, microtubules, intermediate filaments, and septins are four main cytoskeletal components of vertebrate cells (Box 1), yet their roles in autophagy are not fully understood. This Pearl revisits our current understanding of autophagy-cytoskeleton interactions and highlights new concepts and emerging roles for the cytoskeleton in bacterial autophagy.

**Box 1. The Four Cytoskeleton Components**

A. Actin is a globular, multifunctional protein that forms filaments (Figure 3A). Actin (∼40 kDa) is called globular actin (G-actin) in its monomeric form. To generate filamentous actin (F-actin), chains of actin are polymerized (dependent upon adenosine triphosphate [ATP] hydrolysis) and intertwined in a helix with a diameter of ∼7 nm. Actin filaments are polar, with a plus end (where monomers preferentially assemble) and a minus end (where monomers preferentially disassemble).

B. Microtubules are highly dynamic, tubular polymers found throughout the cytoplasm (Figure 3B). Microtubules are made from 13 parallel protofilaments composed of α-tubulin and β-tubulin heterodimers (tubulin monomers are ∼50 kDa). The αβ-tubulin dimers assemble in a head-to-tail manner producing microtubule polymers with a diameter of ∼25 nm. Microtubules are polar and by a process called “dynamic instability” (driven by guanosine triphosphate [GTP] hydrolysis) can assemble and disassemble at the plus end.

C. Intermediate filaments, a family of proteins encoded by ∼70 genes, are a major structural element of human cells (Figure 3C). Intermediate filament proteins assemble to form a tetrameric subunit, and eight tetrameric subunits associate laterally to form a unit length filament (ULF). Individual ULFs join end to end to form short filaments, and short filaments longitudinally anneal to other ULFs and filaments to form longer filaments. Intermediate filaments have a diameter of ∼11 nm (in between that of actin and microtubules), do not require nucleotide (ATP or GTP) hydrolysis, and are nonpolar because of the antiparallel orientation of tetramers.

D. Septins, a fourth component of the cytoskeleton, are evolutionarily conserved GTP-binding proteins that associate with cellular membranes, actin filaments, and microtubules (Figure 3D). Septin subunits (30–65 kDa) are classified into different homology groups and interact through their GTP-binding domain (called the G interface) and their amino-terminal and carboxy-terminal regions (called the NC interface). Septins from different groups (shown in Figure 3D as different shades of red) form complexes (that form rods of 32–40 nm in length) that assemble end to end into nonpolar filaments. Septin filaments can associate laterally and form bundles, and bundles of septin filaments can form higher-order structures, such as rings (which are ∼0.6 µm in diameter).

## Selective Autophagy of Intracellular Bacteria

**Figure 1:**
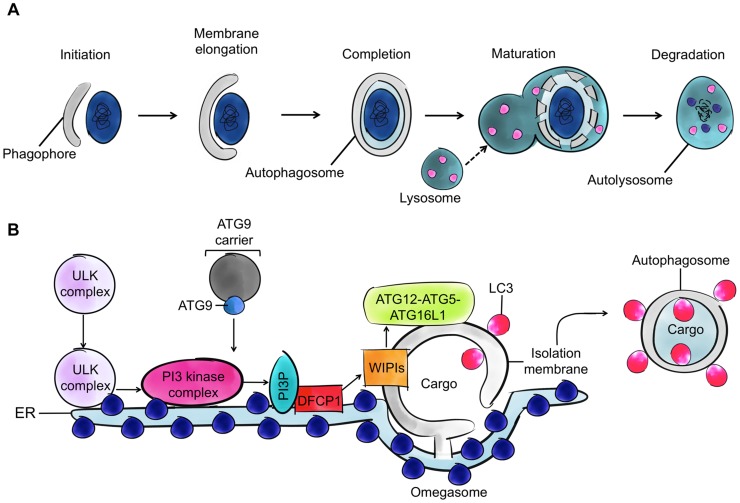
Autophagy and the four cytoskeleton components. A. The process of autophagy. Cytoplasmic material (here a bacterium) is targeted by an isolation membrane (phagophore), and that membrane elongates to form an autophagosome (vacuoles typically 0.3–1.0 µm in diameter). The outer membrane of the autophagosome fuses with the lysosome to form an autolysosome and degrade the enclosed material. B. The molecular events involved in autophagosome initiation, elongation, and completion. The major membrane source is viewed to be the endoplasmic reticulum (ER), and other membrane sources (mitochondria and the plasma membrane) can also contribute. After induction of autophagy, the ATG1-ULK complex translocates to the ER, resulting in activation of PI3 kinase complex and formation of PI3P phospholipid. The formed PI3P phospholipid recruits double FYVE-domain-containing protein 1 (DFCP1) and WD repeat domain phosphoinositide-interacting family proteins (WIPIs), key factors in autophagosome formation. ATG8 family proteins localize to the site of autophagosome nucleation. WIPIs and the ATG12-ATG5-ATG16L1 complex, present on the outer membrane of the isolation membrane, and lipidated ATG8 (ATG8-phosphatidylethanolamine [ATG8-PE]), present on the outer and inner membrane of the isolation membrane, can emerge from subdomains of the ER (called omegosomes).

Autophagy is a membrane trafficking process delivering cytoplasmic material to the lysosome for degradation (Figure 1A). The different steps of canonical autophagy have been well characterized and involve the assembly of at least 36 autophagy-related (ATG) proteins into distinct complexes [5]. The ATG1-UNC-51-like kinase (ULK) complex initiates formation of the isolation membrane (also called a phagophore), the class III phosphatidylinositol 3 (PI3) kinase complex generates PI3-phosphate (PI3P) phospholipid for membrane biogenesis, the ATG12–ATG5–ATG16L1 complex mediates autophagosome formation and elongation, and the ATG8 lipidation system mediates closure of the autophagosomal membrane (Figure 1B). In humans there are six ATG8 orthologues belonging to the light chain 3 (LC3) or γ-aminobutyric acid receptor-associated protein (GABARAP) subfamilies, and by interacting with an extensive repertoire of proteins, they have important roles in mediating membrane-remodelling processes.

Autophagy can be a general, nonselective recycling pathway activated by nutrient limitation and can also degrade cytosolic material, such as intracellular bacteria, in a selective manner. While the exact mechanism of bacterial recognition by autophagy is unknown, ubiquitination is clearly involved [6]. Ubiquitination is a posttranslational modification in which ubiquitin, an 8.5 kDa protein consisting of 76 amino acids, is covalently conjugated to lyseine residues on substrate proteins by three types of enzymes known as E1s (ubiquitin activating), E2s (ubiquitin conjugating), and E3s (ubiquitin ligating). Autophagy receptors (also called sequestosome 1/p62-like receptors [SLRs]), such as p62 and NDP52, recognize ubiquitinated substrates, interact with ATG8 family proteins, and recruit membranes for autophagosome formation. In this way, autophagy can respond to infection by recognizing intracellular pathogens as “nonself” for delivery to the lysosome. However, depending on the pathogen, autophagy can also respond to infection by coordinating cell autonomous signalling and, in some cases, by promoting bacterial replication [2]–[4], [7]. As a result, autophagy is no longer regarded as strictly antibacterial, and the therapeutic potential of autophagy in the treatment of bacterial infection remains unclear.

## Actin Assembly and Autophagy Modulation

**Figure 2:**
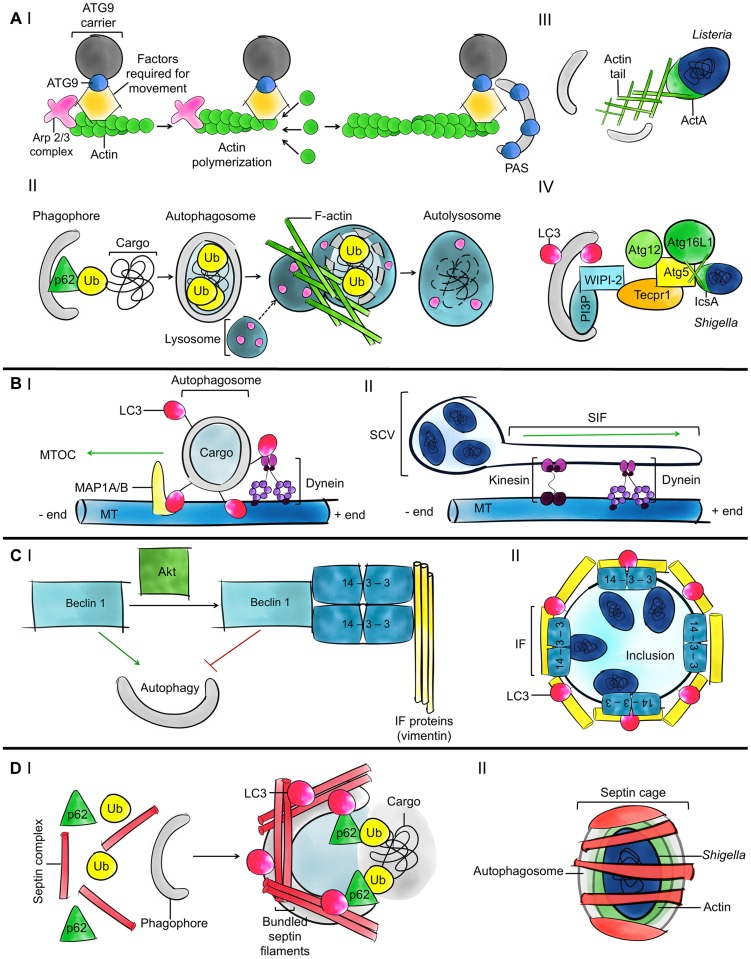
Current understanding of the roles of the cytoskeleton in autophagy. A. Models for the role of actin in autophagy. (I) ATG9 is a transmembrane protein that delivers membrane to growing phagophores. ARP2/3 is a highly conserved seven-subunit protein complex used to nucleate actin filaments and organize them into branched arrays. The polymerization of actin enables the movement of ATG9 to the phagophore assembly site (PAS). (II) In the case of ubiquitin (Ub)-mediated selective autophagy, actin can promote the fusion of autophagosomes and lysosomes to degrade ubiquitinated substrates. (III) After escaping from the phagosome to the cytosol, some bacterial pathogens initiate actin-based motility; most pathogens studied so far promote actin polymerization by using the ARP2/3 complex. Actin polymerization propels the bacteria through the cytosol and into neighbouring cells, allowing them to avoid autophagy. In the case of *Listeria*, actin polymerization by ActA also masks the bacteria from autophagic recognition. (IV) In the case of *Shigella*, actin polymerization by IcsA is strictly required for recognition by ATG5 and bacterial autophagy. When autophagy is induced, PI3P phospholipid associated with phagophores recruits WIPI-2, TECPR1, and ATG5. Damaged mitochondria and protein aggregates can also be recognized by the WIPI-2-TECPR1-ATG5 pathway [16], [17]. B. Models for the role of microtubules in autophagy. (I) Microtubules (MT) and dynein help move autophagosomes from peripheral locations in the cell to the MTOC, where lysosomes are concentrated. ATG8 family proteins could anchor autophagosomes to dynein and transport autophagosomes along the microtubule tracks. ATG8 family proteins could also bind directly to microtubules and by increasing the affinity between microtubules and autophagosomes may facilitate autophagosome trafficking. (II) Following entry into host cells, *Salmonella* are inside a spacious phagosome until the phagosome fuses with lysosomes and shrinks around the bacterium; this compartment is called the SCV. SifA is important for *Salmonella*-induced filament (Sif) formation along microtubules and regulates microtubule-motor (e.g., dynein and kinesin) accumulation on the Sif and the SCV. This regulation may also impact the trafficking of autophagosomes. C. Models for the role of intermediate filaments in autophagy. (I) The phosphorylation of Beclin 1 by the protein kinase Akt promotes a Beclin 1/14-3-3/vimentin complex and inhibits the role of Beclin 1 in autophagy. 14-3-3 proteins are a family of conserved adaptor and scaffolding proteins. (II) Following internalization, *Chlamydia* remodel intermediate filaments (IFs) to form an inclusion and maintain a replicative niche. 14-3-3β localizes in the inclusion membrane. The autophagy machinery is recruited to the inclusion in a cytoskeleton-dependent manner, but the precise role of autophagy in formation and maintenance of the chlamydial inclusion and in bacterial survival is unknown. D. Models for the role of septins in autophagy. (I) Septins may control autophagosome size and shape and also function as cytoskeleton scaffolds on autophagic membranes. Septin filaments are required for efficient recruitment of autophagy critical components including p62 and ATG8 family proteins. (II) When devoid of actin tails, cytosolic bacteria, including *Shigella* and *M. marinum*, can be trapped in septin cages and targeted to autophagy. Septins have been shown to scaffold the autophagy machinery around actin-polymerizing bacteria.

During starvation-induced autophagy, actin has been shown to promote the generation of PI3P for autophagosome formation [8]. In agreement with this, Wiskott-Aldrich syndrome protein (WASP) family member WASH is a nucleation-promoting factor (NPF) for the actin-related protein 2/3 (ARP2/3) complex necessary for the trafficking of ATG9 and autophagosome formation [9]. By contrast, during nutrient-independent, ubiquitin-selective autophagy, actin has been shown to promote the fusion of autophagosomes to lysosomes [10]. Taken together, these observations suggest that actin can link membrane acquisition to autophagosome biogenesis or regulate the fusion of autophagosomes to lysosomes, depending on the cargo targeted for degradation (Figure 2AI, 2AII).

**Figure 3:**
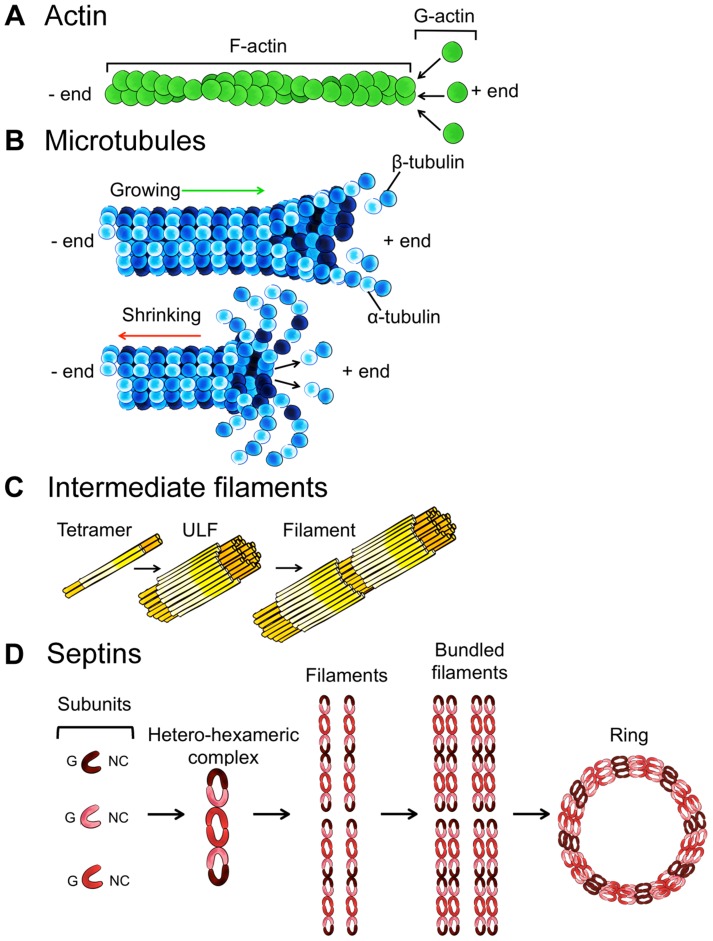
Schematic overview of the four cytoskeleton components. A. Actin assembly into filaments. B. Microtubule assembly and disassembly. C. Intermediate filament protein assembly into filaments. D. Septin complex, filament, and ring assembly.

Some bacterial pathogens, including *Listeria monocytogenes* and *Shigella flexneri*, polymerize the host actin cytoskeleton for actin-based motility and intracellular spreading [11]. Proteins involved in actin-based motility have been linked to autophagy. However, work has shown that *Listeria* and *Shigella* are recognized by different autophagy pathways, and the precise role of actin in autophagy differs between pathways (Figure 2AIII, 2AIV). In the case of *Listeria*, the polymerization of actin by ActA (a bacterial effector that acts as an NPF for the ARP2/3 complex) prevents the recruitment of ubiquitin, autophagy receptors, and ATG8 family proteins, thereby inhibiting autophagosome formation [12], [13]. In the case of *Shigella*, the polymerization of actin by IcsA/VirG (a bacterial effector that binds the host NPF neuronal-WASP [N-WASP]) is strictly required for recognition by ATG5 and the tectonin domain-containing protein TECPR1 to promote autophagosome-lysosome fusion [13]–[17]. These observations suggest that the polymerization of actin can inhibit or promote bacterial autophagy and that the role of actin in autophagy differs between pathogens and/or NPFs. Further work using intracellular pathogens that form actin tails, including also *Rickettsia* in the spotted fever group, *Burkholderia pseudomallei*, and *Mycobacterium marinum*, will help to identify and characterize the diverse roles of actin in autophagy.

## Microtubules and Autophagosome Trafficking

Microtubules facilitate autophagosome trafficking and are viewed to promote autophagosome biogenesis and the fusion of autophagosomes with lysosomes [18]. Dynein, a motor protein, is required for autophagosome trafficking along microtubules. To interact with autophagosomes, dynein can bind to ATG8 family proteins and their interaction partners, microtubule-associated protein 1A and 1B (MAP1A and MAP1B). In this way, autophagosomes can move along microtubule tracks toward lysosomes concentrated near the microtubule organizing centre (MTOC) (Figure 2BI) [19].

Several pathogenic bacteria can modulate the dynamics of microtubules during infection [20], and it is likely that pathogen interference with microtubules has a profound impact on autophagy. *Listeria* ActA can recruit LaXp180/CC1, a binding partner of the microtubule-sequestering protein stathmin [21]. An ActA-LaXp180-stathmin interaction could destabilize microtubules, prevent autophagosome trafficking, and facilitate bacterial actin-based motility. *Shigella* VirA is a type III secretion system (T3SS) effector protein that can destroy surrounding microtubules and help actin-based motility [22]. VirA also exhibits guanosine triphosphate-activating protein (GAP) activity and can manipulate Rab1, a guanosine triphosphatase (GTPase) required for autophagy of *Salmonella* [23], to suppress autophagy [24]. The microtubule network and motors play a crucial role in maturation of the *Salmonella*-containing vacuole (SCV), and the T3SS effector SifA is essential for this process (Figure 2BII) [25]. Without a stable SCV, a significant fraction of *Salmonella* can become cytosolic and targeted to autophagy, resulting in either restriction [26], [27] or promotion of bacterial replication [28]. To mediate anti-*Salmonella* autophagy, the F-BAR-containing protein transducer of CDC42-dependent actin assembly (TOCA-1, also called formin-binding protein 1-like or FNBP1L) interacts with ATG3, a ubiquitin-like conjugating enzyme essential for ATG8 lipidation, and promotes autophagosome biogenesis [29]. By contrast, in the case of *Shigella*, TOCA-1 helps bacteria to avoid recognition by autophagy markers [30] and to polymerize actin tails by relieving the autoinhibited state of N-WASP [31]. In summary, microtubules promote vesicle trafficking required for autophagy and can be targeted by bacterial pathogens for intracellular survival. Future work investigating the role of microtubule dynamics in the maintenance of pathogen-containing vacuoles and in counteracting actin-based motility will help to clarify the different roles of microtubules in delivering bacteria to autophagy.

## Intermediate Filaments and Autophagy Stabilization

Intermediate filaments are more stable and exhibit less dynamic behaviour than actin filaments or microtubules. In agreement with this, vimentin intermediate filaments have recently been shown to suppress autophagy dynamics by interacting with Beclin 1/ATG6, a protein crucial for autophagy initiation [32]. The phosphorylation of Beclin 1 by the protein kinase Akt promotes a Beclin 1/14-3-3/vimentin complex and inhibits the role of Beclin 1 in autophagy, highlighting a fundamental link between autophagy and intermediate filaments (Figure 2CI).

The rearrangement of intermediate filaments during bacterial infection is not as well characterized as in the case of actin or microtubules. *Chlamydia trachomatis*, an obligate intracellular bacterial pathogen, replicates within a large vacuole called an inclusion. 14-3-3β was the first eukaryotic protein found to interact with the chlamydial inclusion [33]. Recent work has shown that *Chlamydia* can remodel intermediate filaments and actin to provide structural integrity to the inclusion and also to prevent cytosolic detection of inclusion contents (Figure 2CII) [34]. Interactions between the chlamydial inclusion and autophagy have been described, and localization and function of the autophagy machinery (e.g., LC3, MAP1A, and MAP1B) at the chlamydial inclusion is clearly cytoskeleton associated, involving the actin, microtubule, and intermediate filament networks [35]. However, the role of autophagy in the restriction or promotion of *Chlamydia* replication is not fully defined. In accordance with a role for intermediate filaments in autophagy stabilization, ATG8 family proteins are not lipidated into the inclusion membrane, and chlamydial inclusions are characteristically not targeted to autophagy. These data suggest that pathogens that manipulate intermediate filaments for intracellular survival can also manipulate autophagy. To counteract this, members of the guanylate-binding protein family (GBPs, a family of GTPases induced by interferon-γ) can potentiate an antibacterial effect of autophagy through their ability to stimulate fusion of chlamydial inclusions with autophagosomes [36].

## Septin Assembly and Autophagy Promotion

Septins have been mostly implicated in cytokinesis, a process in which autophagy is tightly regulated, and their role in nondividing cells is poorly understood [37]. The recruitment of autophagy critical components, e.g., p62 and LC3B, to their sites of function can be reduced by septin depletion [14], suggesting a general role for septins in autophagic activity (Figure 2DI). Septins and the septin-like GTPase of immunity-associated proteins (GIMAPs) function as nucleotide-regulated scaffolds on intracellular membranes, and GIMAP6 interacts with GABARAPL2 for recruitment to autophagosomes [38]. The specific interaction between GIMAP6 and GABARAPL2, similarly to what has been described between NDP52 and LC3C [39], suggests different roles for different ATG8 family proteins during autophagy and may highlight another level of specificity underlying selective autophagy.

During *Shigella* infection, septins entrap actin-polymerizing bacteria in cage-like structures that restrict motility and dissemination (Figure 2DII). Experiments have shown that septin cages prevent actin-tail formation and target bacteria to autophagy [13], [14]. Septin caging has been observed for *Shigella* and *M. marinum* but not for *Listeria* because ActA masks the bacteria from autophagic recognition (see above). These observations indicate that septin cages assemble in certain pathways of actin polymerization, for example, actin polymerization by WASP family proteins (as occurs in the case of *Shigella* and *M. marinum*), and/or that a cytosolic source of membrane is required to facilitate septin assembly surrounding the bacterium. In turn, septins may control the shape of autophagic membranes, as shown for phospholipid-based liposomes in vitro [40]. It has also been shown in vitro that ARP2/3 preferentially binds curved actin filaments [41]. Septins influence actin filament curvature [42] and may have a key role in biasing branched actin networks to mediate bacterial autophagy. Whether bacterial pathogens can exploit septin assembly to counteract autophagy is not known, yet work using zebrafish (*Danio rerio*) has suggested that septin cages are an important defence mechanism to clear intracellular bacteria in vivo [43].

## Concluding Remarks

Studies using intracellular bacterial pathogens have shown great potential to significantly advance our understanding of autophagy-cytoskeleton interactions. Despite new insights, many outstanding issues remain. For example, the precise signals targeting bacteria to autophagy and the source of membrane for autophagosome biogenesis (endoplasmic reticulum, mitochondria, and/or plasma membrane) are poorly understood. Work has shown that intracellular bacteria can trigger multiple canonical and noncanonical (i.e., alternative autophagy pathways independent of some core machinery components) autophagy pathways [2]. However, what determines the pathway of autophagy responding to bacterial infection is not yet clear. To help resolve these issues, future studies investigating intracellular pathogens and how they modulate the cytoskeleton during infection shall be crucial to illuminate structural determinants of autophagy and their roles in mediating disease outcome. They can also reveal new links between autophagy and the cytoskeleton.

Finally, autophagy dysfunction has been implicated in various human diseases including cancer, neurodegenerative diseases, inflammatory diseases, and infectious diseases. Cytoskeleton dysfunction has also been implicated in many of these diseases. Could autophagy dysfunction underlie the pathogenesis of particular cytoskeleton disorders (and vice versa)? An in-depth understanding of autophagy-cytoskeleton interactions in vivo will be critical to fully appreciate how autophagy and the cytoskeleton can function in immunity and bacterial clearance and if autophagy-cytosketon interactions can be therapeutically manipulated for human health.
